# Melanotic Schwannoma of the Vagina: A Report of a Very Rare Tumor and Review of the Literature

**DOI:** 10.1155/2019/8521834

**Published:** 2019-06-17

**Authors:** Kofi Effah, Stefan Seidl, Edith Gorges, Patrick Kafui Akakpo

**Affiliations:** ^1^Battor Catholic Hospital, North Tongu District, Volta Region, Ghana; ^2^22459 Hamburg, Frohmestrasse 59, Hamburg, Germany; ^3^Department of Pathology, School of Medical Sciences, University of Cape Coast, Ghana

## Abstract

Melanotic schwannoma (MS) is a rare nerve sheath tumor with fewer than 200 cases reported. MS has uncertain malignant potential and comprises 1% of all nerve sheath tumors with a predilection for the spinal nerve roots. An even rarer location for this tumor is the vagina. Up to 55% of MSs that contain psammoma bodies are associated with the Carney complex, an autosomal dominant syndrome. Criteria for malignancy in MS are still not well established and long term follow-up of patients is recommended. A 26-year-old woman presented with a bleeding vaginal tumor which was diagnosed as MS following excision. The clinical, histopathological, and immunohistochemical features of this tumor are discussed.

## 1. Background

Melanotic schwannoma (MS) is a rare nerve sheath tumor composed of Schwann cells, some of which are pigmented [1, 2]. MS is thus considered a variant of schwannoma, though the clinical and histopathological features of the two lesions differ greatly. To date, fewer than 200 cases of MS have been reported worldwide and they account for less than 1% of all nerve sheath tumors [1]. MS has a predilection for intracranial structures and spinal nerve roots [1, 2]. There is no sex predilection. Histopathologically, up to 50% of MSs contain psammomatous calcification with 50% of these psammomatous MSs being linked to the Carney complex. Though most MSs are benign tumors, about 10% are malignant and have a tendency for local recurrence and metastasis [1–3]. Clinically, 50% of these tumors are associated with Carney complex (an autosomal dominant syndrome) and patients have characteristic patchy skin pigmentation, myxomas, and endocrine tumors of the adrenal cortex, pituitary, testis, and thyroid [1–3]. A rarer case of MS of the vagina is presented and the literature reviewed.

## 2. Case Presentation

**Figure 1:**
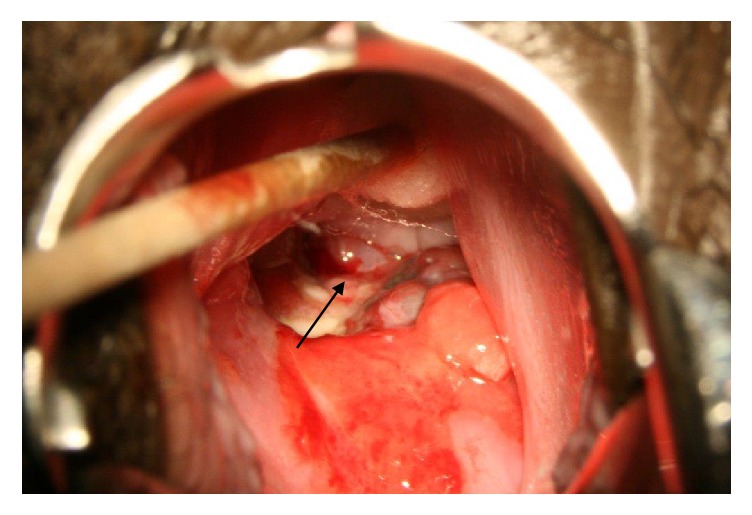
A view of the tumor (arrow) at initial speculum examination prior to excision with cervix not visualized because the tumor was in the way.

A 26-year-old Para 0+0 woman presented to our facility in December 2008, complaining of prolonged, heavy bleeding of two weeks' duration during her last menstrual period. This was the first such episode. In addition, she also had episodes of postcoital bleeding. She had not missed her menses and a pregnancy test was negative. General examination was clinically normal with no significant findings. A speculum examination revealed a polypoid lesion in the upper vagina measuring 4cm across. The cervix was not distinctly seen (Figure 1). The initial impression was a cervical tumor to rule out malignancy. An abdominal ultrasound showed a normal uterus with no masses within the uterus.

**Figure 2:**
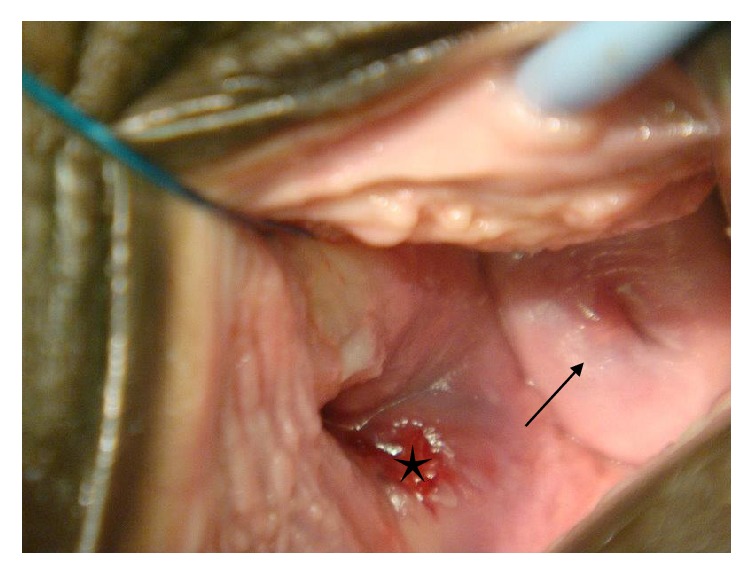
Showing the cervix (arrow) and adjacent residual tumor in the vagina (star) after the first incomplete excision.

Histopathological examination of an incision biopsy done on January 7^th^ 2009 suggested a blue nevus with a differential of schwannoma. The patient was subsequently counselled for an examination under anaesthesia and excision of the tumor. The initial excision was incomplete with subsequent colposcopic examination showing a residual 2.5cm tumor in the posterior vaginal wall. The adjacent cervix was now visible and was normal (Figure 2, arrow). Final excision of the residual tumor with free margins confirmed by histopathological examination was performed on August 28, 2009, two months after the incomplete excision of the tumor. This showed a mass entirely located in the vagina measuring 6cm in its widest diameter. Our patient had an uneventful postsurgical period and was discharged home on postoperative day three.

## 3. Histopathological Examination

**Figure 3:**
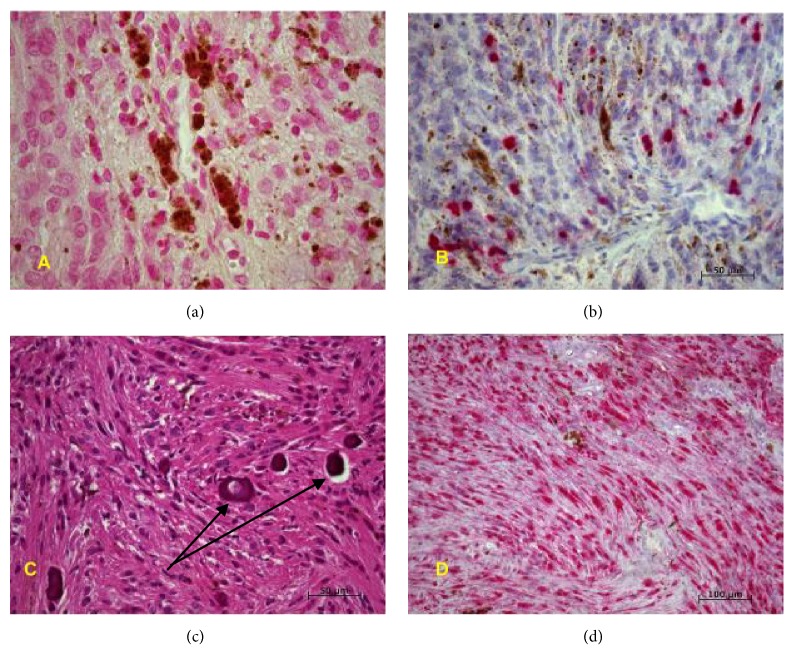
(a) Negative Turnbull's blue histochemical stain for iron, (b) MIB-1(Ki-67) immunohistochemical stain showing low proliferative index, (c) Hematoxylin and Eosin stain showing psammoma bodies (arrows), (d) positive S-100 immunohistochemical stain.

Three specimens were presented at different times with the last one being the reexcision of the residual tumor. Though the initial haematoxylin and eosin stained sections suggested a blue nevus, this diagnosis was made awaiting excision. Following excision, the tissue received was identical in morphology to the first albeit with more prominent fibrillary and reticular areas. The cells were of intermediate size and were round or spindle shaped, containing nuclei of intermediate to large size with small nucleoli. There were scattered large cells with nuclei atypia. However, no mitotic figures were seen. There were repeated clusters of cells containing granules of brown melanin pigment that were negative for iron stain (Figure 3(a), negative, Turnbull histochemical stain). In addition, there were scattered melanophages. Immunohistochemically, the Ki-67 proliferation index was low (10%) (Figure 3(b)). There were scattered psammoma bodies within the tumor (Figure 3(c), arrows). The tumor was S-100 protein positive (Figure 3(d)). There were no areas of necrosis. There were foci of chronic inflammatory change dominated by lymphocytes. A final diagnosis of psammomatous melanotic schwannoma (MS) (WHO grade I) was made. The tumor was completely excised at the last excision. It was mentioned in the histopathology report that MS is associated with Carney complex and neurofibromatosis and had the ability to metastasize on occasion. Long term follow-up of the patient was recommended.

A more thorough physical examination showed no evidence of Carney syndrome or neurofibromatosis. Cervical screening with the HPV DNA testing on February 15, 2019, was negative for high risk HPV.

## 4. Discussion

MS occurs among a wide range of ages but typically occurs in young adults with a mean age of 38 years. Our patient was 26 years. There is no sex predilection. Though MS may occur anywhere in the peripheral nervous system, they most commonly occur in the paraspinal sympathetic chain and the gastrointestinal tract with the esophagus and the stomach being the commonest sites in the gastrointestinal tract. Very rarely, they have been found in the cerebellum, orbit, heart, trachea, bronchus, cervix, bone, soft tissue, and skin [1]. In our patient, this tumor occurred in the vagina. We were unable to find a report of vaginal MS in the English literature after a search of PubMed central and Google Scholar, though cases of regular schwannoma have been reported in the vagina [4]. The clinical presentation of MS varies depending on its location and our patient presented with vaginal bleeding. When located in regions related to nerves, patients may present with sensory and motor abnormalities and up to 35.5% of patients present with symptoms indicative of involvement of spinal nerves or other nerves [1]. Our patient did not have any such symptoms.

Histopathologically, MS may be of psammomatous or nonpsammomatous type. Our patient's MS was of the psammomatous type and these are known to more frequently involve autonomic nerves of viscera. Again, up to 50% of these are associated with Carney syndrome/complex (CC), an autosomal dominant syndrome that is characterized by, among other things, spotty pigmentation of the skin, cardiac, mammary, and uterine myxomas, primary pigmented nodular adrenocortical disease with Cushing's syndrome, growth hormone producing pituitary adenoma, Sertoli cell tumors of the testis/ovaries, thyroid adenomas, and breast adenomas [1–3]. Another genetic condition associated with MS is neurofibromatosis type I, also an autosomal dominant condition that is characterized by the presence of neurofibromas and café au lait spots [1]. Both syndromes have mutations on chromosome 17 though at different loci: 17q11 for neurofibromatosis type I and 17q24 for CC. Our patient did not have any lesions to suggest that she had CC or neurofibromatosis type I and after 10 years still has none. In addition, she is without local recurrence or metastasis. Our patient did not have any genetic testing. Nonpsammomatous MSs occur sporadically and usually affect spinal nerves and paraspinal ganglia. These nonpsammomatous MSs are not associated with CC or neurofibromatosis.

Histopathologically, this tumor's features were identical to those described in the literature [1]. Histopathological characteristics that suggest malignancy in MS have not been clearly outlined and agreed on due to the rare nature of the tumor and the poor correlation between histopathological features and behavior [1, 5–7]. However, the presence of lesional cells with large, vesicular nuclei that have macro nucleoli, brisk mitotic activity, and foci of necrosis suggests aggressive behavior [1, 5]. In one study, it was concluded that only mitotic count greater than 2 per 10 high power fields correlates with metastasis [1, 6, 7]. Our patient's tumor lacked these features and also showed a low Ki-67 proliferation index. It was thus classified as a low grade tumor. Follow-up of the patient was however still recommended without the need for any other treatment modality; this is because MSs have been known to show late metastasis even though they may have an initial benign clinical appearance [1, 6–8]. In this regard, 10% -35% of MSs even when devoid of overt histologic atypia may follow a malignant course with a recurrence rate of 18.2% and a metastatic rate of 9.1% after a mean follow-up period of 5.9 years reported [2, 3, 8, 9]. The essence of long term follow-up can therefore not be overemphasized and is strongly advised as reported by Decouvelaere* et al*. who found that only 53% of the 77 patients they reviewed were disease-free after a follow-up period of more than five years [10]. Ten (10) years after our patient's tumor was excised, she is still well with no evidence of recurrence or of Carney syndrome. Due to the rare nature of this tumor, postsurgical management is controversial and large tumors, incompletely resected tumors, and tumors showing aggressive features may require radiation and chemotherapy in conjunction with surgery [1, 8–12].

## 5. Conclusion

To our knowledge, this is the first case of MS of the vagina that has been reported. In line with the clinicopathological features of MSs at other reported sites, they are of uncertain malignant potential and may be associated with CC and neurofibromatosis. Their correct diagnosis ensures appropriate management with adequate excision and long term follow-up.
